# Foreign Correspondence

**Published:** 1885-04

**Authors:** A. Lagorio

**Affiliations:** No. 6 Piazza Nazionale, Chiavari, Italy


### FOREIGN CORRESPONDENCE.

*To the Editors of the* CHICAGO MEDICAL JOURNAL AND EXAMINER—*Gentlemen:* In some of my correspondence, which you have been so kind as to publish, I have alluded to several new remedies now in vogue in many European hospitals, and which I thought might be of some interest and value to your readers. Having myself paid considerable attention to the subject of therapeutics, I always hail with joy the appearance of new acquisitions to our armamentarium. Kairine and antipyrine are already well known as our latest valuable antipyretics, but a third one has just come forward, and is called *Talline.* This new alkaloid is a base of chinoline, which Kraup has recently prepared by synthesis. The name of talline is given, because when treated with perchloride of iron or any other oxidizing powder, it forms green crystals. Talline, when treated with tartaric, sulphuric and hydrochloric acid, forms salts very soluble in water, possessing strong antifermentative properties, but especially antipyretic, which action has been recently studied by Jacksch, of Vienna, (*Gaz. Med de Paris*). He ascribes to it a very potent febrifuge action. When given near a paroxysm of intermittent fever, talline prevents the development of the febrile stage, as it also abbreviates its duration if given during it. But it will not act as a cure, the paroxysm will return, so that quinine is needed and must be given to complete the cure. Talline will produce rapid lowerings of temperature when given in the course of pneumonia, of acute articular rheumatism, of erysipelas, of puerperal fever, and of tuberculosis. After the administration of $\frac{1}{4}$, $\frac{1}{2}$ or $\frac{3}{4}$ of a gram the febrile temperature is lowered many degrees in four or five hours, and will not return to the primitive degree for some time. It has no effect as a medicament on the disease. The remedy is eliminated by the kidneys without causing any albuminuria nor glycosuria. The urine acquires a brownish tint, which changes into green when the liquid is examined by transparency. The following advantages have been ascribed to talline over antipyrine: There is less sweating during the stage of defervescence, the rigors are less frequent when the temperature is rising; the vomiting, cyanosis, collapse are always absent. It possesses a not disagreeable bitter-aromatic taste.

Another new preparation is *Ozoneine*, which Inimus has presented to the Biological Society of Paris. It is a liquid, and is prepared by Bech, a pharmacist of Paris. Ozoneine is a condensation of ozone, or a solution of it in water. Very highly disinfectant properties are attributed to it. Ozone was largely experimented with by Brand, with success, in the wards of choleraics and variolous patients at Toulon, and its efficacy was proven by Guiol, Long, Peyrremond and Rey-Escudier. Long and Guiol asserted that the significant immunity observed on those attending the cholera patients must be attributed to the ozonized air supplied continuously in the wards. (*Riv. Clin. e Terap.*) Ozonized water was used long ago by Lender in inflammations, in gangrene, glaucoma, neuralgia, diabetes and in all infective diseases, especially in diphtheria, for its local action in destroying the pseudo-membranes, which action sometimes occurs very rapidly. To disinfect wards of hospitals with ozone, there is a powder prepared by Krebs, Kroll & Co., of Berlin, composed of peroxide of manganese, permanganate of potassium and oxalic acid. Two tablepoonsful of this powder with two tablespoonsful of water are sufficient to generate enough ozone to disinfect a hall for two hours. No metals must be left exposed.

Cocaine is still the leading topic of discussion in all medical circles. Since writing my last letter, many new observations have been presented. Innumerable are the indications for its use. But I will not usurp any more space in your journal to present to you all the reports, especially those of Mazza, Grasulli, Rosmini, Ielenk, DeMagri, etc., etc., in the diseases of the eye, and of Loughi, Desarenes and others, in diseases of the ear, for they all agree in pronouncing it a valuable remedy. By some opthalmologists it is used as a substitute for atropine. So far nothing else has been written about it except in praise.

Cremation is gradually progressing and becoming familiar throughout Italy. Education and persuasion are the only ways by which this reform must enter the minds and the hearts of the people. For this reason the progress it has made may seem slow, but it is not prudent to use violence in the control of the sentiments and prejudices to impose a funereal usage which is destined in time to become universal. One of the greatest obstacles to cremation was the deficiency of suitable furnaces which could rapidly, and with little expense, cremate the dead. But to-day the problem is solved, for it has been clearly demonstrated, that with the modern furnaces, cremation can be adopted as one of the greatest sanitary reforms. Italy has been the inaugurator of this method of disposing of the dead, and so far she has maintained her primacy over all nations in this field. The city of Milan has two cremation furnaces, one of Goviano and the other of Venini; Lodi, Rome, Cremona, Varese have adopted the Govini's furnace; Padua, Udine, Brescia, Novara have Venini's furnace, recently modified and which work to perfection. Florence, Pisa and Como, have also the Venini furnace. Leghorn has adopted Spasciani system. At Messina, and in the Variguano Lazaretto, near Spezzia, engineer Guzzi, by order of the Government, has built a furnace which combines the value of the Govini system and of the Venini process. During last year there have been 113 cremations, 82 males, 31 females. Two bodies were brought from France. In three an autopsy was previously made. Eight were exhumed. One was a priest. Four were the bodies of choleraics (at Cremona).

On the 15th of January, Dr. Lewis Nincini performed Porro's operation (utero-ovarian amputation) on a woman forty years old, primipara, at full term and twelve hours in labour. She had a narrow pelvis, caused by rickets. The operation lasted fifty-five minutes, and was finished without the least inconvenience. There was no spray, but the most rigorous antiseptic dressing was used. The skillful and valiant surgeon in this difficult operation was equal to his reputation, having operated with that security and ability for which he is noted. The patient soon rallied from the effects of chloroform and the shock of the operation, and is doing well. The new-born, a female, is alive and well.

The Secretary of State, Signor Mancini, has proposed to the foreign powers an International Sanitary Congress to convene in Rome for discussing and adjusting international sanitary measures against cholera. Premier Depretis will present to the Parliament a bill regarding the measures to be taken in case of epidemic diseases, and determining the duties of governors, sanitary councils and mayors.

With a philanthropic intent to better the conditions of the farming classes, especially in those provinces where pellagra is constantly present, the Minister of Agriculture, Industry and

Commerce has offered eight gold medals with 500 francs each, and eight silver medals with 300 francs each, to the promotors, founders and exercisers of economical ovens for the use of the rural population; or of any other institution indicated to better the condition of farmers; also eight gold and silver medals for the best colonical houses. The applications for the concours must be presented not later than the 31st of July, 1885. It is indeed time that something be done to eradicate this horrible disease, which annually takes away hundreds of human beings. What the poor classes want is: less taxes and better food. The government is throwing away millions of dollars in African expeditions and colonial acquisitions, while the money is most needed at home to relieve the pauperism so prevalent in the pellagra districts.

Money and provisions are the *antiseptics* of pauperism and famine, which are the only and true *bacilli* of pellagra.

A. LAGORIO, M. D.

No. 6 Piazza Nazionale, Chiavari, Italy.

